# Ultrasound-Identified Trigger-Point and Ultrasound-Guided IncobotulinumtoxinA (Xeomin®) Injection for Refractory V2–V3 Trigeminal Neuralgia: A Case Report

**DOI:** 10.7759/cureus.98146

**Published:** 2025-11-30

**Authors:** Yeonsik Jang

**Affiliations:** 1 Aesthetic Medicine, Cheong-Ayeon Clinic, Seoul, KOR

**Keywords:** botulinum toxin, incobotulinumtoxina, intradermal injection, trigeminal neuralgia, ultrasound-guided botulinum toxin

## Abstract

Trigeminal neuralgia (TN) can be refractory to systemic anticonvulsants. Targeted botulinum toxin type A (BoNT-A) injections are an emerging local therapy. Ultrasound (US) may help identify subclinical peripheral trigger points that are not evident on palpation. We report a case of a 50-year-old male with longstanding left-sided V2-V3 TN who experienced rapid and durable pain relief after US-identified trigger-point mapping, followed by combined intradermal and US-guided intramuscular injections of incobotulinumtoxinA (Xeomin®). On clinical examination, trigger points were not prominent; however, real-time ultrasonography (Clarius L20) identified subtle focal fasciculations/tremor and hyperdynamic areas consistent with peripheral trigger sites. Treatment comprised intradermal “skin-botox” microinjections along the mandibular/zygomatic line (total 20 U; left 15 U, right 5 U) using 34G × 4 mm needles, and US-guided intramuscular masseter injections (total 50 U; 30G × 25 mm needles; higher proportion to symptomatic left side). Pain improved from VAS 8/10 → 2-3/10 within one week and remained reduced for approximately three to four months. No adverse events were observed. The patient also reported aesthetic improvement of jawline contour. US can detect subclinical trigger-point activity and guide precise BoNT-A delivery. Incorporating US-identified trigger-point mapping and US guidance (Clarius L20) allowed targeted intradermal and intramuscular incobotulinumtoxinA injections that produced durable analgesia and cosmetic benefit in refractory TN. Prospective studies should evaluate standardized US-based protocols.

## Introduction

Trigeminal neuralgia (TN) is characterized by paroxysmal facial pain along one or more divisions of the trigeminal nerve. Although anticonvulsants such as carbamazepine or gabapentin remain first-line, many patients experience suboptimal relief or intolerable side effects. Botulinum toxin type A (BoNT-A) has emerged as a promising minimally invasive alternative, with randomized trials and systematic reviews reporting beneficial effects in TN [1-4].

Ultrasound (US) offers real-time visualization of soft tissue anatomy and dynamic features. Recent case series and technique reports describe the value of US for mapping trigger points and guiding injections in facial pain syndromes [5-7]. In this case, we used a high-frequency handheld probe (Clarius L20) both to identify subclinical trigger points - areas showing subtle tremor/fasciculation not palpable on examination - and to guide intramuscular masseter injections.

For clarity, in this manuscript, we use the following operational terms: "fasciculation" - a brief, spontaneous focal twitch of a muscle fascicle visible on high-frequency US; "hyperdynamic motion" - localized increased tissue motion observed on real-time imaging; and "ultrasound-identified trigger zone" - a focal site where US shows fasciculation or hyperdynamic motion that correlates temporally and spatially with the patient’s symptoms and was selected for targeted injection. We emphasize that this represents an operational working definition for the purposes of targeting and reporting in this case and does not equate fully with classical myofascial trigger point diagnostic criteria (taut band, spot tenderness, and referred pain).

## Case presentation

Patient information and history

A 50-year-old male presented with a multi-year history of left-sided facial pain consistent with TN involving the maxillary (V2) and mandibular (V3) divisions. Prior management included long-term gabapentin/pregabalin with incomplete symptom control (baseline VAS 8/10). The patient elected to pursue localized injection therapy for pain control and simultaneous cosmetic improvement of jawline contour.

Consent and ethics

Written informed consent for the procedure and for publication of de-identified images and clinical data was obtained. Per institutional policy, this single case report did not require formal IRB review; patient consent documentation is on file.

Pre-procedure assessment and US mapping

On physical examination, focal trigger points were not clearly palpable. Dynamic US mapping using a handheld high-frequency device (Clarius L20) was then performed. During gentle jaw movement and at rest, US revealed subtle focal fasciculations/tremor and localized hyperdynamic motion within superficial tissue along the left mandibular and zygomatic line, consistent with peripheral trigger-point activity. These ultrasonographically identified sites guided the superficial intradermal microinjection pattern. The masseter muscle bulk and depth were assessed sonographically to plan the intramuscular needle trajectory and depth [6].

We defined a US-identified trigger zone operationally as a focal area in which real-time high-frequency US demonstrated brief, spontaneous focal fasciculation or localized hyperdynamic motion that correlated with the patient’s site of maximal symptoms. These observations were captured on video and reviewed. We interpreted these sonographic signs conservatively and correlated them with the patient’s reported point tenderness or symptom reproduction during dynamic maneuvers. We note that formal validation of sonographic criteria against independent standards (e.g., EMG or intraoperative correlation) is limited.

Methods/injection technique

Product: IncobotulinumtoxinA (Xeomin®)

Reconstitution and lot numbers were recorded in the clinical chart (Table 1).

**Table 1:** Summary of injection sites, techniques, and dosages used during the ultrasound-guided botulinum toxin treatment.

Area	Route/depth	Needle	Total units (Xeomin®)	Distribution/notes
Masseter (bilateral)	Intramuscular (US-guided)	30G × 25 mm	50 U (total)	Left 35 U / Right 15 U; real-time ultrasound guidance
cheek (V2) Jawline (V3)	Intradermal microinjections	34G × 4 mm	20 U (total)	Left 15 U / Right 5 U; microdroplet technique

Ultrasound Device

The Clarius L20 handheld linear probe was used for (1) dynamic mapping of superficial trigger points (identifying subtle tremor/fasciculation), and (2) real-time guidance for intramuscular masseter injections [5-7].

Needles and Volumes

**Figure 1:**
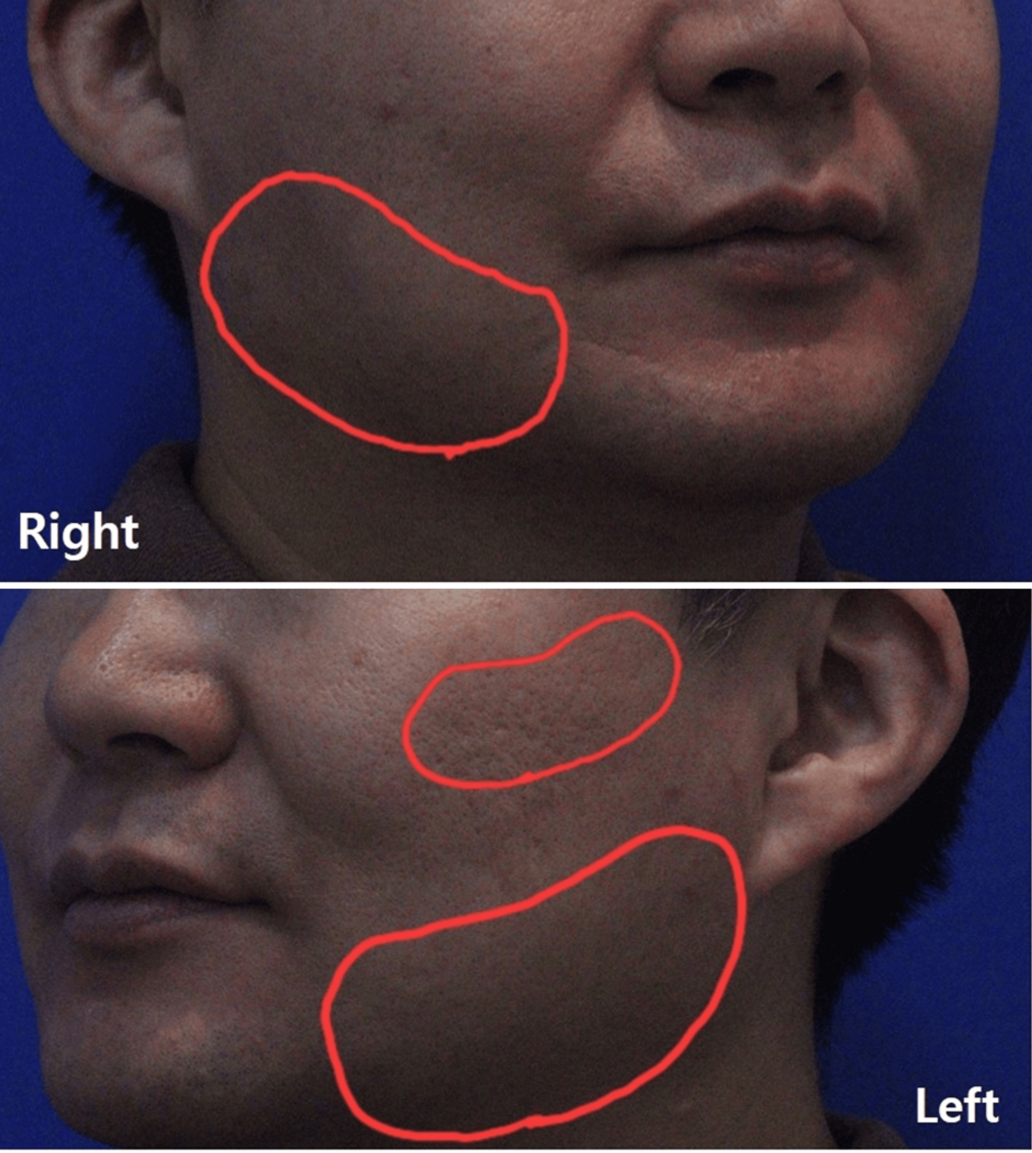
Intradermal (skin-botox) microinjection sites along the jawline and zygomatic regions.

Intradermal (skin-botox) microinjections: 34-gauge × 4 mm needles: Total superficial dose = 20 U incobotulinumtoxinA (left 15 U; right 5 U), divided across ~10-12 microinjection points along the jawline/zygomatic distribution, placed into the papillary/upper reticular dermis (Figure 1).

**Figure 2:**
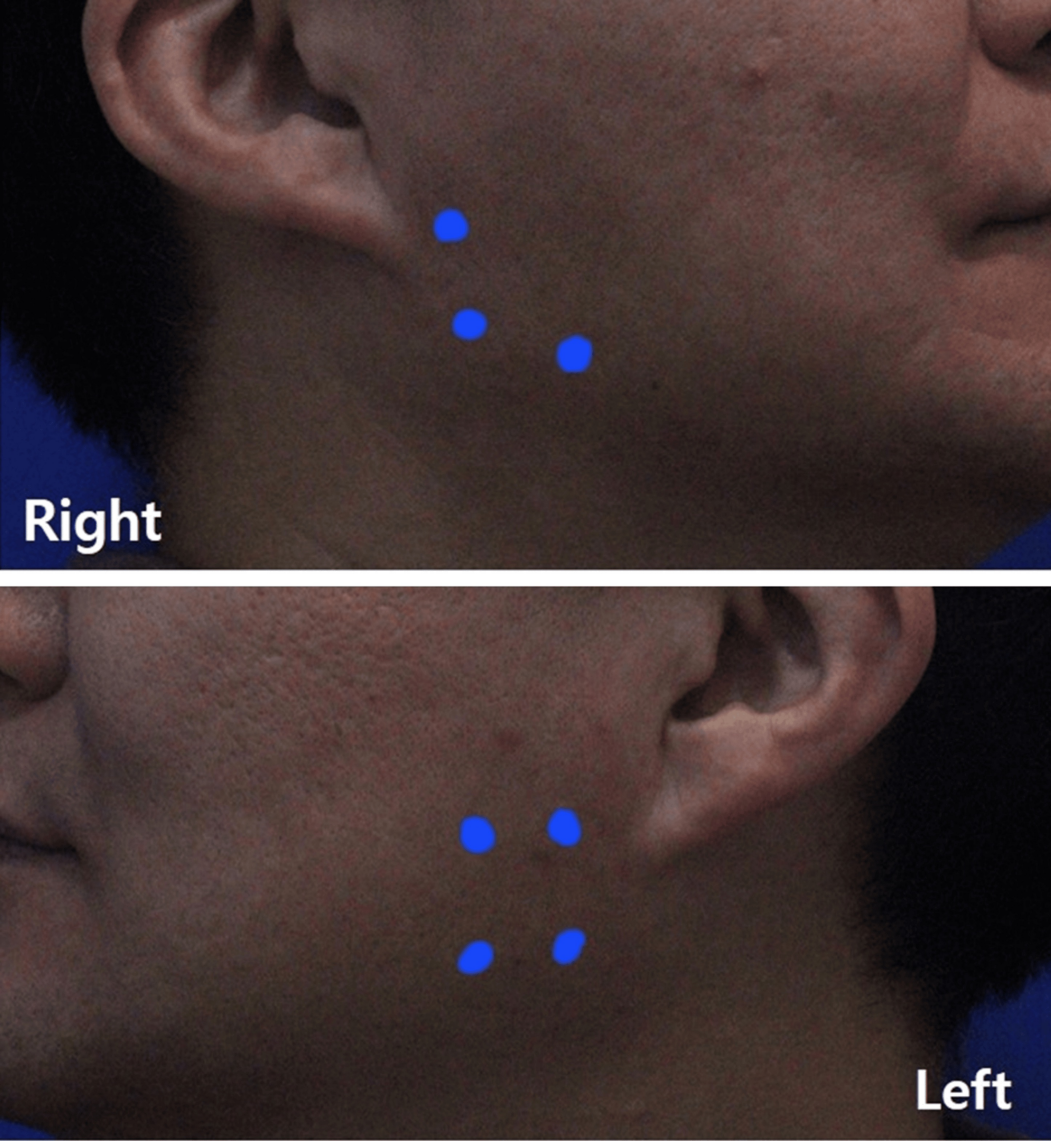
Intramuscular injections site into the bilateral masseter muscles.

Intramuscular (masseter) injections: 30-gauge × 25 mm needles were used for masseter intramuscular injections under real-time US visualization. A total of 50 U of incobotulinumtoxinA was administered, with 35 U injected on the left side and 15 U on the right side under real-time US guidance (Figure 2).

Dose allocation favored the symptomatic side (eg, 35 U left versus 15 U right intramuscular; 15 U left versus 5 U right intradermal) based on greater symptom intensity, patient-reported pain distribution, and larger masseter bulk on clinical and sonographic assessment. This pragmatic, symptom-guided asymmetric dosing strategy follows clinical practice patterns where dosing is titrated to lateralized symptom burden and muscle mass; exact dosing was individualized to balance efficacy with safety.

Technique

Skin Antisepsis and Sterile Field as per Clinic Protocol

For intradermal microinjections, 34G × 4 mm needles were used to deposit microdroplets (0.02-0.05 mL) superficially at mapped trigger points and along the mandibular/zygomatic line. Care was taken to avoid intravascular injection.

For intramuscular masseter injections, an in-plane approach under real-time US guidance was used. Needle tip visualization was confirmed prior to each aliquot. Aspiration was performed prior to injection (Videos 1, 2). 

**Video 1:** Masseter fasciculation captured by Clarius L20 ultrasound imaging.

**Video 2:** Ultrasound-guided botulinum toxin injection at the masseter fasciculation point captured by Clarius L20 ultrasound imaging.

Post-procedure observation was done for 30 minutes; discharge with routine aftercare instructions.

Results/clinical course

The patient reported improvement in pain to VAS 3/10 within one week and to VAS 2-3/10 by two weeks, with symptomatic relief maintained for approximately three to four months. No procedure-related adverse events occurred (no facial weakness, dysphagia, dysarthria, or sensory deficit). The patient also reported subjective aesthetic improvement of jawline contour and high satisfaction. Follow-up visits at one and three months documented ongoing benefit. No changes were made to baseline oral medications (gabapentin/pregabalin) during the observation period.

## Discussion

This case highlights several clinically relevant points.

US can detect subclinical trigger-point activity. In our patient, trigger points were not conspicuous on palpation but were identified as focal areas of subtle tremor/fasciculation and hyperdynamic motion on dynamic US (Clarius L20). Such sonographic signs provided objective mapping to guide superficial microinjections. Similar US-based approaches and guided injection techniques have been described [5-7].

Combined superficial and deep targeting may be synergistic. Intradermal microinjections likely target superficial nociceptive terminals and cutaneous trigger zones, whereas intramuscular masseter injections may reduce myofascial contribution and deeper nociceptive input. The combination - delivered precisely using US mapping and guidance - yielded rapid and durable analgesia in this refractory case. Prior randomized and observational studies support analgesic effects of BoNT-A in TN [1-3].

Safety and precision are enhanced with US guidance. US allowed safe, in-plane visualization of needle trajectory for masseter injections and informed choice of injection depth and needle length, reducing the theoretical risk of inadvertent intravascular or peri-neural injection. Reports on BoNT-A use in orofacial pain and temporomandibular disorders further highlight the balance between efficacy and risk [8-10].

Mechanism

BoNT-A inhibits peripheral release of nociceptive mediators (substance P, CGRP, and glutamate) and may reduce peripheral and central sensitization, providing a biologic explanation for the analgesic effect. In addition to peripheral effects, recent experimental and review literature describes mechanisms that may include retrograde axonal transport of toxin-related signals and subsequent modulation of central pain processing (central desensitization). These mechanisms may help explain prolonged analgesia beyond the expected peripheral action window and are discussed in recent mechanistic reviews. [11]

Limitations

Single-case uncontrolled design; operator-dependent mapping and dosing; lack of objective electrophysiologic confirmation of the sonographic findings; and absence of long-term follow-up beyond the reported three to four months. Nonetheless, US-identified trigger-point mapping combined with US-guided injections provides a reproducible, documentable approach suitable for protocolized evaluation. We acknowledge that using real-time US to identify focal fasciculations or “hyperdynamic” foci currently lacks standardized diagnostic criteria; imaging settings, probe frequency, recording duration, and operator interpretation can vary between centers and may affect reproducibility. Development and validation of objective sonographic criteria (e.g., standardized probe frequency, minimal observation time, and explicit definitions distinguishing fasciculation from other motion artifacts) are necessary for wider generalizability.

## Conclusions

In this single case of refractory V2-V3 trigeminal neuralgia, ultrasound-identified focal fasciculations were used to guide targeted intradermal and intramuscular incobotulinumtoxin A injections, resulting in rapid and sustained pain relief with high patient satisfaction and no significant adverse events. While promising, these findings derive from a single, uncontrolled observation and therefore require cautious interpretation. The report highlights the potential value of dynamic ultrasound mapping for improving injection precision and suggests that future prospective studies should validate sonographic criteria, standardize targeting protocols, and incorporate objective outcome measures. The author recommends larger, controlled investigations to determine reproducibility, optimal dosing strategies, and long-term efficacy and safety.
